# An Empirical Analysis on the Impact of Innovation Network Structure on Crossover Innovation Performance of Emerging Technologies

**DOI:** 10.1155/2022/8312086

**Published:** 2022-07-31

**Authors:** Yanxi Jin, Xing Cao

**Affiliations:** ^1^Business School, Central South University, Changsha 410083, China; ^2^School of Economics and Trade, Hunan University of Technology, Zhuzhou 412007, China

## Abstract

The crossover innovation springing up in emerging technologies has drawn wide attention from scholars. Innovation network, as an effective way for major innovation-driven entities towards less relevant risks and higher efficiency, can significantly affect the crossover innovation performance. This paper analyzes the evolution law of the innovation network of autonomous driving technology based on the Social Network Analysis (SNA) and by using the data on joint applications for invention patents of such technology during 2006–2020. Furthermore, the structural eigenvalues of the network evolution are calculated for the regression analysis of the relationship between network structure and crossover innovation performance. The empirical results show that network centrality, structural hole, and relationship intensity have a positive effect on crossover innovation performance of emerging technologies, while network clustering has a negative effect. Emerging technology enterprises should constantly improve their technological innovation ability, improve their status and influence in the innovation network, establish cooperation with appropriate innovation partners, further expand their own technical knowledge fields, and obtain innovation resources by optimizing the network structure so as to enhance the crossover innovation performance.

## 1. Introduction

With the increasingly complex and changing environment of technology and market, it is difficult for major innovation-driven entities to meet the needs of technological innovation only by their own limited resources, so they cooperate with partners to exchange resources and promote technological innovation in order to gain competitive advantages in the market, thus creating formal or informal innovation networks among the nodes [1]. Innovation network, a basic institutional arrangement for cooperation and communication among the nodes, can effectively promote transmission and transfer of technological knowledge within the network [2, 3]. Therefore, innovation networks of integrated resources have become an important choice for major innovation-driven entities to avoid risks, improve innovation efficiency, and promote technological innovation. Innovation network is a dynamic integral whole, where the overall network structure as well as the location, heterogeneity, resource control, and connection relationship of the nodes will change with the continuous interactions between major innovation-driven entities, promoting its continuous evolution. The structure of the innovation network is crucial to technological innovation performance of the nodes in the network [4]. Kim et al. believed that rational innovation network structure promotes diversified knowledge acquisition and heterogeneous resource sharing among major innovation-driven entities through communication and learning, which improves the technological innovation performance [5]. Xie and Wang found through empirical researches that network structure affects enterprises' absorption ability and then innovation performance [6].

In 1994, the Wharton School's Huntsman Research Center developed and implemented the “Emerging Technology Management Research Program,” which first introduced the concept of emerging technologies, defining them as science-based technologies that can create or change an industry [7]. The breeding and development of emerging technologies is different from the traditional path-dependent technology development, which is marked by major technological breakthroughs and convergent innovations, embodied as a process of ever-increasing various necessary resources, and is featured by high complexity, ambiguity, and market uncertainty different from the traditional technology R&D. With the introduction of the concept of emerging technologies, academics have paid increasing attention to issues related to emerging technologies and conducted rich research. Particularly in recent years, the fast-changing new round of scientific and industrial revolution has witnessed collaborations highlighted by digital and smart features among emerging technologies, such as digital manufacturing, the Internet, biological electronics, new materials, and new energy, making crossover innovation a more prominent hot topic [8] by international scholars. Vandermerwe and Rada pointed out that crossover innovation is manifested in the fact that some enterprises have started to provide users with integrated solutions, including manufacturing products, rather than only physical products, thus making the boundaries of industries with originally obvious industrial boundary characteristics gradually blurred [9]. From the perspective of industrial development, Greenstein believed that the characteristics of crossover innovation are manifested in the contraction or disappearance of industrial boundaries once applied to industrial growth [10]. Grimpe and Sofka believed that crossover innovation of emerging technologies is a behavior where major innovation-driven entities share technological and market resources beyond certain limits with counterparts for better innovations [11]. According to Tao et al. [12] and Zhang and Ren [13], crossover innovation was a comprehensive innovation from thinking to action. Emerging technology enterprises reorganized themselves by integrating their own capabilities and potential resources beyond organizational, industrial, or familiar fields to achieve all-round and multilevel innovations. Zhang et al. held that crossover innovation was an innovation strategy for enterprises to break technological and industrial boundaries, integrating functions and revolutionizing products [14]. Based on existing researches, this paper concludes that the crossover innovation of emerging technologies refers to those emerging technology enterprises aiming to develop emerging technologies and effectively facilitate the deep fusion and effective coupling of knowledge of emerging technologies and original technologies through communication and cooperation with different major innovation-driven entities [15], so as to break through the original knowledge boundaries and innovation barriers to create new technological knowledge and result in new technologies, products, or industries.

Current researches on innovation network focus on its overall evolution or capture characteristics with a few enterprises as the object using simulation, case, and other research methods, which lacks sufficient data. The empirical research studies on how innovation network structure impacts enterprise technological innovation are fruitful, but few pay attention to crossover innovation. Moreover, in terms of research methods, most related researches adopt questionnaires for structural equation analysis, which is difficult to reflect the innovation performance characteristics generated by the “crossover” of enterprise technology. Thus, using the data on joint applications for invention patents in the emerging technology field of autonomous driving during 2006–2020, this paper analyzes the evolution law of the innovation network of such technology by the SNA and studies the influence of network structure changes on the crossover innovation performance of the nodes. Autonomous driving technology, which achieves disruption through crossover integration, is an emerging technology in the process of crossover innovation. The research in this paper has practical inspirational value for the continued innovation of such technology as well as for crossover innovation in emerging technologies of a similar nature in the future, which particularly has important theoretical value and guiding significance for major innovation-driven entities of emerging technologies on how to formulate effective cooperative innovation strategies and how governments build innovation cooperation platforms in the construction of innovation networks to promote the long-term development of emerging technologies.

## 2. Theoretical Basis and Research Hypothesis

The crossover innovation of emerging technologies refers to the creation of new technologies and knowledge from the interaction and fusion of original different technologies, with the crossover and heterogeneity of knowledge connection as its intrinsic characteristics [16]. Innovation network, an effective institutional arrangement for major innovation-driven entities to improve efficiency, enables these entities to access knowledge and resources in a convenient and efficient way, which is an important premise for crossover innovation [17]. This paper uses patent data and identifies the technical fields involved among nodes according to the International Patent Classification (IPC) and then measures the crossover innovation performance of the nodes in quantity. Also, the location, heterogeneity, degree of resource control, and connection relationship of the nodes will change with the network, thus showing differences [18] in crossover innovation performance. This paper, therefore, selects the indicators of network centrality, structural hole, relationship intensity, and network clustering to measure the network structure characteristics of the nodes so as to analyze the relationship between innovation network structure and crossover innovation performance of emerging technologies.

### 2.1. Network Centrality and Crossover Innovation Performance

The status and location of the nodes in the network can be measured by centrality that represents their influence [19, 20]. The nodes with high centrality have greater influence and wider influence range, control more network resources, and lead the communication and cooperation among the nodes. The quality and speed of their technological innovation activities can also better adapt to the environment. Emerging technologies characterized by greater risks and long R&D cycle have higher requirements for enterprises' own resources and adaptability to the external environment. If an emerging technology enterprise has a high position in the network and can mobilize more heterogeneous knowledge and technical resources, it is easier to cross existing technology fields and generate better crossover innovation performance [21]. Therefore, higher network centrality means better crossover innovation performance. Based on the above analysis, this paper proposes the following hypothesis:

  H1: network centrality has a positive effect on crossover innovation performance of emerging technologies.

### 2.2. Structural Hole and Crossover Innovation Performance

Given the large number of nodes in the network, “all nodes are connected” is an ideal state. In most cases, some nodes have few connections, resulting in a “network hole,” namely, a structural hole [22]. The nodes occupying the structural hole have monopolistic advantages of heterogeneous resources and high network powers in the network, making them easy to obtain more network resources more quickly at lower costs, and further more development advantages [23]. Fleming and Mingo Chen verified the above-mentioned innovation advantages [24], including easier access to external resources for crossover technological innovation activities, greater opportunities for technological development, and better fulfillment of technological innovation ability to improve the crossover innovation performance. The essence of crossover innovation of emerging technologies is heterogeneous knowledge fusion, which often occurs in the intersections of different technological fields [25]. The enterprises occupying the structural hole are more likely to gain heterogeneous resources, effectively reduce invalid connections with other organizations, and carry out deeper technological innovation activities for efficient crossover innovation [26], better performance, and strong drive to upgrade certain fields to core technologies through continuous investment. Based on the above analysis, this paper proposes the following hypothesis:

  H2: structural hole has a positive effect on crossover innovation performance of emerging technologies.

### 2.3. Relationship Intensity and Crossover Innovation Performance

Relationship intensity reflects the frequency of connection between the nodes in the network. The difference in relationship intensity will affect the communication and cooperation and information transmission among the nodes, thus having an important impact on innovation performance. When the relationship intensity is low, less time and emotion involved weaken trust between the nodes, which hinders the dissemination of tacit knowledge and the sharing of heterogeneous resources, thus reducing the complementarity and utilization of resources of both partners. As the relationship intensity improves with greater scope and frequency of communication and cooperation among the nodes, opposite results happen, which facilitates the dissemination of tacit knowledge and the sharing of heterogeneous resources [23] and the crossover fusion of technology and knowledge, along with crossover innovation realization and crossover innovation performance [27–30]. Based on the above analysis, this paper proposes the following hypothesis:

  H3: relationship intensity has a positive effect on crossover innovation performance of emerging technologies.

### 2.4. Network Clustering and Crossover Innovation Performance

Network clustering refers to the degree to which a pair of relationships in the network is surrounded by a common third party, reflecting how closely the nodes connect with each other [31]. The crossover innovation of emerging technologies is a process of deep fusion and effective coupling among different technologies and knowledge as well as the creation of new knowledge and technologies. The higher level of the network clustering, the deeper the interaction between network members [32], which promotes the dissemination and sharing of knowledge in different technological fields among the major innovation-driven entities. The cross-boundary and cross-field interaction, fusion, and reorganization between emerging technologies and the original ones create new knowledge, resulting in crossover innovation [33]. Based on the above analysis, this paper proposes the following hypothesis:

  H4: network clustering has a positive effect on crossover innovation performance of emerging technologies.

## 3. Research Design

### 3.1. Data Source and Processing

Joint patent application is the recognition of technological innovation cooperation between joint applicants, which can reflect the fusion of technological knowledge in patent cooperation. The development of such cooperation innovation network through joint patent application has been widely recognized by the academic circle. Patent is one of the important carriers of technological information, including invention patent, appearance design patent, and utility model patent. Among them, the invention patent has a high technical level and originality, which can well measure the applicant's technological innovation ability. Content including the patentee (innovation subject), technical connection, cooperative relationship, and citation has been widely used in the empirical researches related to innovation network, technological diffusion, and innovation performance.

Autonomous driving technology is an intelligent vehicle technology developed on the basis of computer technology and has been used in the market since the beginning of this century. It uses a variety of technologies such as artificial intelligence and GPS systems to work together to enable cars to drive unmanned and follow instructions issued by a computer. According to the Emerging Technology Maturity Curve published by Gartner (the world's most authoritative information technology research and advisory firm), autonomous driving technology is rated as one of the most promising emerging technologies and has formed a crossover innovation network with the participation of many complementary players from upstream and downstream industries and different technologies and industries in corporate practice. This network shows a relatively complete and continuous dynamic evolution process from the initial exploration of autonomous driving technology to the gradual improvement and value realization of the technology, which provides a good research context for exploring how to realize crossover innovation of emerging technologies through innovation networks [34]. Based on this, this paper explores the mechanism of the role of innovation network in promoting crossover innovation in emerging technologies by collecting data on joint applications for invention patents of autonomous driving technology.

This paper uses the Social Network Analysis (SNA) to construct an innovation network. The SNA is a network science analysis method based on the knowledge of statistics, mathematics, graph theory, computer, and other disciplines. It provides the idea of network analysis based on two main elements, relationship and structure, which is now widely used in academia for network-related research [35] and thus is an effective tool for analyzing emerging technological innovation networks. In this study, two types of powerful Social Network Analysis software, Gephi 9.2 and Ucinet 6.0, were used for visual analysis and quantitative measurement of innovation network.

The data for this article were obtained from the Derwent Innovation Index (DII), a widely used database containing a large amount of complete patent data of high authority [36]. This paper used the advanced retrieval of DII for data collection. Few patent data before 2006 were of little research value and would lag behind due to time-consuming patent application and authorization, but that before 2020 was novel and could reflect the overall development trend of autonomous driving technology, so the time span was 2006–2020. The search strategy was based on the keywords related to autonomous driving in the topic sphere (TS), and the information retrieval expression was TS = “autonomous vehicle^*∗*^” OR “driverless car^*∗*^” OR “self-piloting automobile” OR “self-driving car^*∗*^,” with^*∗*^ as a wildcard to retrieve the basic variants of word cells. Finally, a total of 9,527 patents were collected on April 9, 2021, the date for data retrieval. Data selecting and processing step by step were necessary due to the huge amount and redundant information.

#### 3.1.1. Conversion of Data Format

The fields required for the exported pure text data were extracted, including patent number, patent application date, title, applicant, abstract, IPC, and patent citation. The invention patents with two or more enterprises rather than individuals as the patentees were selected because the objects were mainly enterprises, scientific research institutions, and other organizations in the field of autonomous driving technology. Through data selecting and processing [37], the data format consistent with the imported Gephi was finally generated for network visualization analysis.

#### 3.1.2. Division of Study Time Window

Innovation network evolves accompanied by network structure changes, and its evolution can be reflected by network structure characteristics. This paper divides the evolution stages of autonomous driving technology by using the rolling method. It often takes an enterprise several years of continuous technological innovation activities to apply for an invention patent, so it was believed that three years could effectively reflect the sustainability of technological innovation activities [38]. Therefore, this paper divided the network evolution into five stages with three years as a rolling window period.

#### 3.1.3. Development of the Cooperative Innovation Network

**Figure 1:**
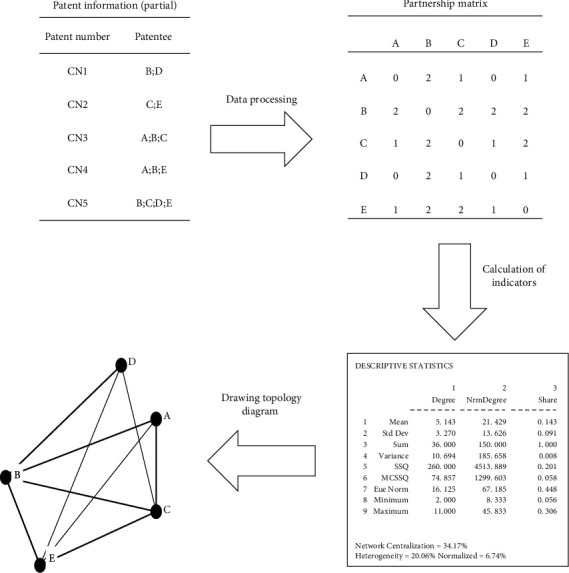
Schematic diagram for modeling of cooperative innovation network.

Joint invention patents contain at least two joint patentees. If two or more patentees jointly own one patent, there is a cooperative relationship between them, and the number of patent items applied jointly represents the cooperation times between them. The statistics of data on joint patent applications were transformed into the matrix of partnerships. In the matrix, the number of patents in cooperation is indicated by a number; if there is no cooperation, the matrix is filled with “0.” The partnership matrix was then imported into the Ucinet software to calculate network structure metrics and also into the Gephi software to generate the cooperative innovation network topology diagram. The node in the diagram represents the patentee, the connecting line represents the joint application relationship between joint patentees, and the thickness of the connecting line is the number of patent items applied jointly, which represents the amount of cooperation between joint patentees, as shown in Figure 1. The establishment of cooperative relationship is usually accompanied by the tacit technological knowledge flow, affecting the technology fusion and crossover innovation [39–41].

### 3.2. Data Analysis

#### 3.2.1. Application for Autonomous Driving Patents

**Figure 2:**
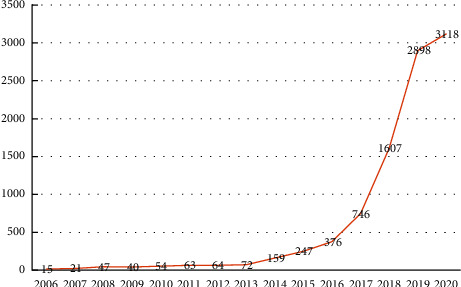
Analysis of the total number of invention patent applications for autonomous driving technology.

The number change of patent applications can reflect the trend of R&D investment, market prospect, and technology development process. During 2006–2013, the increase was slow until greater one in 2014, especially a big jump after 2016, as shown in Figure 2. In general, the total number of invention patent applications for autonomous driving technology showed an upward trend during 2006–2020, with the average annual applications above a certain level.

#### 3.2.2. Evolution Graph of the Cooperative Innovation Network

**Figure 3:**
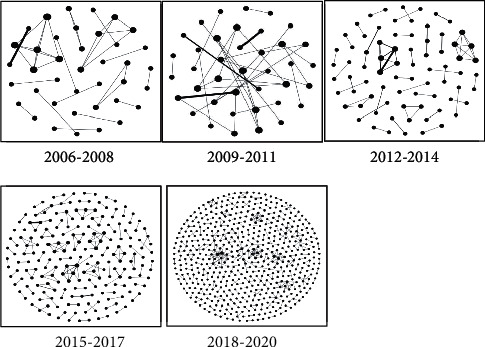
Cooperative innovation network graphs.

As shown in Figure 3, the cooperative innovation network graphs, with the patentee as the node and the cooperative relationship between the joint patentees as the connecting line, were drawn according to the five stages.

The establishment of cooperative relationships among major innovation-driven entities in the network is usually accompanied by the flow of technological knowledge and the exchange and sharing of heterogeneous resources, thus promoting the crossover integration of technologies and resulting in crossover innovation [42]. It can be seen from the figure that the size of the cooperative innovation network among various organizations in autonomous driving technology has gradually expanded since 2006, with increased nodes and connecting lines year by year. Particularly after 2015, both of them increased significantly. It showed that the cooperation in autonomous driving technology was more common, and the major innovation-driven entities favored technological innovations through extensive crossover cooperation, making autonomous driving technology gradually cover more and more different major innovation-driven entities and technology fields so that the overall technology network rapidly integrates, absorbs external knowledge, and continuously expands, indicating that the development of autonomous driving technology is a crossover innovation process of continuous convergence and integration of different technologies among different major innovation-driven entities and evolves over time.

#### 3.2.3. Evolution Characteristic Analysis of the Cooperative Innovation Network

This paper adopted the statistical indicators of density, average degree, average path length, and clustering coefficient proposed by Albert and Barabási [43] and Freeman [44], which have been widely used to analyze the structure and properties of the network, as shown in Table 1.

Calculations and comparative analysis of the network indicators in the five stages revealed significant changes in the overall network structure over time, as shown in Table 2. Compared with slowly increasing network size before 2015, during 2015–2017 and 2018–2020, with the gradual development of cooperative innovation activities, the number of major innovation-driven entities and cooperative relationship in autonomous driving technology rose significantly, indicating that the number of major innovation-driven entities and technologies involved in autonomous driving technology was increasing and the network was gradually more open. The network density reduced from 0.046 to 0.003, which reflected that the cooperative innovation network of autonomous driving technology transformed from high to low density. Moreover, the average clustering coefficient and the degree of network clustering decreased, which indicated that the degree of network monocentricity was weakening and the centrality of nodes was gradually decreasing, indicating that cooperation among major innovation-driven entities and technology integration was no longer limited to certain specific key technology fields, further indicating that network connectivity was enhanced and crossover integration of different technology fields among different major innovation-driven entities was becoming more frequent. The average degree and average path length increased after a slight drop during 2012–2014, which meant that the number of major innovation-driven entities cooperating and exchanging with a certain major innovation-driven entity was increasing, which further indicated that the phenomenon of crossover integration of technologies among different major innovation-driven entities was becoming more and more common.

During 2006–2008, 2009–2011, and 2012–2014, the vast majority of subnetworks in the cooperative innovation network were always with close membership and a regular structure based on social or geographical connections. At this time, there were fewer major innovation-driven entities in the network, but the degree of clustering was high. The long-term stable cooperative relationship among them facilitated the accumulation of technological knowledge in a certain field in the network and prevented such knowledge from being spread beyond the network.

Once the technological knowledge amounts to a breakthrough, the network can build the initial technology chain. Therefore, the first three stages were to generate autonomous driving technology. During 2015–2017 and 2018–2020, the density of the network further reduced, the clustering decreased, and the average degree together with average path length increased, indicating more frequent cooperation among different major innovation-driven entities and more openness. Further increase in the number of major innovation-driven entities and cooperative relationship contributed to a large number of incremental innovations and the outward extension of technology chain. At the same time, the enhanced heterogeneity of major innovation-driven entities in the network accelerated the flow and transmission of diversified technological knowledge in the network and facilitated the crossover fusion and innovation among different technologies, thus driving the rapid development and evolution of autonomous driving technology in recent years.

Based on the above analysis, it can be seen that the development of autonomous driving technology was accompanied by the continuous expansion and openness of cooperative innovation network, and the network presented evolutionary characteristics such as more heterogeneous major innovation-driven entities, universal cooperation, richer and more diversified technological knowledge resources, and gradually blurred network boundaries were constantly breaking through the restrictions of geography, industry, or technological field, which fully reflects the structural characteristics of the evolution of innovation network in the process of crossover innovation of emerging technologies. With the continuous improvement of technology level and the further enhancement of emerging technology development requirements, the geographical, industry, and technology boundaries of the autonomous driving technology innovation network will be further broken.

### 3.3. Variable Design

#### 3.3.1. Dependent Variable

Measuring the technological innovation performance through invention patent data can objectively reflect the technological innovation level of major innovation-driven entities and avoid the possible social desirability in the scale survey, which has already gained global recognition. Crossover innovation performance of emerging technologies reflects the innovation output efficiency of emerging technology enterprises in cross-border cooperation R&D. Referring to the method of using the number of patents to reflect innovation performance in current researches and based on the IPC, this paper measured the crossover innovation performance of emerging technologies by counting the number of invention patents cooccurring with IPC numbers. The IPC (International Patent Classification) is an internationally used tool for classifying and searching patent documents. Each patent has its own IPC number, which reflects the technological field involved in each patent. If the same patent has more than one classification number, it means that the patent covers different types of technological fields and generates cooccurrence. Therefore, if there were two or more different IPC numbers in the same patent simultaneously, this patent could be defined as the result of crossover fusion of technologies and resulted in crossover innovation performance. In addition, the total number of invention patents included invention patents separately developed and authorized by enterprises, as well as invention patents jointly applied and authorized by enterprises and other patent owners.

#### 3.3.2. Independent Variable

*(1) Network Centrality*. Centrality can measure how central the nodes are in the network, including degree centrality, between centrality, and betweenness centrality. Among them, the degree centrality, or the number of other network nodes directly connected to a specific node, is the most intuitive, so it is most commonly used to evaluate node centrality. The degree centrality reflects the connection relationship between a specific node and other nodes in the network, as well as the communication and cooperation ability of the nodes in the network relationship. The higher degree centrality of a node indicates that the node is more central in the network and more active in the network relationship, namely, more powerful and influential.

The nodes with higher degree centrality always have easier access to heterogeneous resources necessary for innovation from the network environment, thus generating crossover innovation performance more easily. Since the degree centrality of the nodes is not comparable under different network sizes, the centrality index of individual network, namely, the relative degree centrality, was selected for variable measurement. The calculation formula was as follows:

(1) $$\begin{array}{c} N_{RD}\left(i\right)=\frac{N_{AD}\left(i\right)}{\left(n-1\right)}, \end{array}$$

where *N*_*R*  *D*_(*i*) is the relative degree centrality of node *i*; *N*_*A*  *D*_(*i*) is the number of other nodes directly connected to node *i*, namely, the absolute centrality of node *i* in the network; *n* is the total number of network nodes, namely, the network size.

*(2) Structural Hole*. Structural hole describes the structural location of the nodes in the network, which highlights the nonredundant connection among the nodes and emphasizes the important role that nodes play in the network connection relationship. Burt held that the nodes occupying the structural hole were more advantageous for accumulating and controlling a variety of necessary and nonredundant heterogeneous resources for innovation [22]. The existing researches measure the structural hole from four dimensions, specifically efficiency, effective scale, hierarchy, and restrictiveness. Among them, the effective scale describes the nonredundant connection relationship among the nodes, which means that the individual network size of the nodes removes the redundancy among individual nodes. Based on previous researches, the structural hole of the nodes was measured by the effective scale in this paper. According to Burt's structural hole theory, the measurement formula was as follows:

(2) $$\begin{array}{c} SH_{i}=\sum_{j}\left(1-\sum_{q}P_{iq}M_{jq}\right),\;q\neq i,j, \end{array}$$

where *SH*_*i*_ is the structural hole of the node *i*; *j* is all the nodes in the network connected to the node *i*; *q* is each third-party node in the network other than *i* or *j*; *P*_*iq*_*M*_*jq*_ is the redundancy between the node *i* and a specific node *j*; *P*_*iq*_ is the proportion of the relationship of the actor node *i* input to *q*, representing the marginal strength of the node *i* input relationship.

*(3) Relationship Intensity*. Relationship intensity (NS_i_) reflects the communication and cooperation among different nodes, which also measures the frequency and tightness of connections among the nodes during a specific period. The nodes with higher relationship intensity can obtain and use resources more easily from the network to carry out innovative activities. Former researches mainly measure the relationship intensity by the number of interactions, connection frequency, depth and width of cooperation, degree of trust, and stability and persistence of relationships [45–48]. Based on that, the relationship intensity was measured by calculating the average amount of cooperation among the nodes over a fixed period.

*(4) Network Clustering*. Clustering coefficient represents the degree of clustering or close connection among the nodes in the network, which is to measure the efficiency of the network structure. Previous researches show that nodes with frequent interaction are easier to form a closely connected network group. Compared with the random version, the innovation network with a high clustering coefficient can better promote cooperative innovation among different entities and its performance improvement.

The clustering coefficient includes local and global parts. Among them, the local clustering coefficient describes the degree of clustering near each node in the network. As this paper mainly studies enterprises, the local clustering coefficient was adopted. The calculation formula was as follows:

(3) $$\begin{array}{c} NC_{i}=\frac{2E_{i}}{k_{i}\left(k_{i}-1\right)}, \end{array}$$

where *E*_*i*_ is the actual number of edges between the *k*_*i*_ adjacent nodes of node *i*; *k*_*i*_(*k*_*i*_ − 1)/2 is the maximum number of possible edges between the *k*_*i*_ adjacent nodes of the node *i*. If *k*_*i*_ = 0 or *k*_*i*_ = 1, *E*_*i*_ = 0. At this point, *NC*_*i*_ was 0.

#### 3.3.3. Control Variable

The crossover innovation performance can be affected by many factors, including the innovation network environment and the technological resource type (TRT) involved in emerging technology enterprises. Referring to Lerner's researches [49], this paper used the top four letters or digits of IPC to measure the TRT, namely, subclass, which can reflect patents' technological fields and possible application scope. In addition, the TRT invested each year was measured by inquiring about the top four letters or digits of IPC of enterprises' invention patents.

The evolution of the innovation network is accompanied by the continuous entry and exit of the nodes; hence, the sample data of this study belonged to panel data under an equilibrium state. Further statistics found that there were few cooperative patents before 2016, but booming explosively until recent years, which was in line with the actual development of autonomous driving technology. Thus, the influence of the TRT and the time invested by enterprises were taken into consideration and analyzed as the control variables.

## 4. Empirical Analysis

### 4.1. Sample Data Analysis

**Figure 4:**
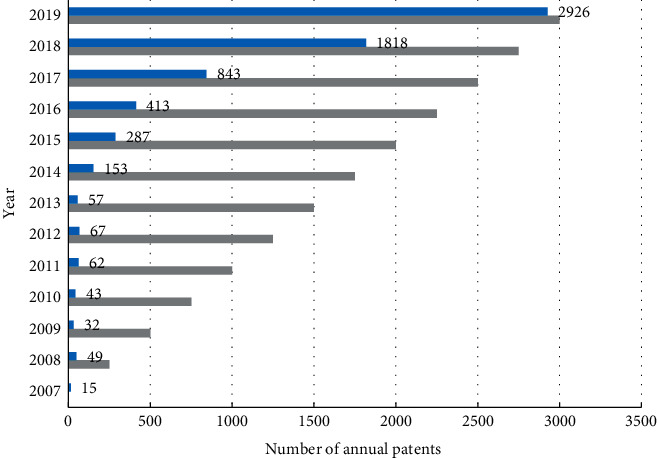
Applications for invention patents for autonomous driving technology.

Through further screening and statistics of the collected data on invention patents, a total of 6,765 invention patents for autonomous driving technology (including at least two main IPCs) were selected out during 2007–2019, involving 2,096 patentees, as shown in Figure 4. Before 2014, the number of patent applications was growing slowly. The economic downturn in 2008 led to a decline in the number of patents in 2009, then a slow climb, and afterwards a small decline during 2012–2013. Since 2014, the total invention patent applications have increased sharply from 153 to 2,926 in 2019, showing a rapid and steady growth trend. This indicates that the innovation effect of crossover fusion of autonomous driving technology has become more and more prominent with technology development in recent years.

Based on the researches of Deeds and Hill [50], the number of joint patent applications in a certain year is regarded as the result of continuous cooperative innovation among the major innovation-driven entities. Taking three years as the duration of cooperative relationship, the joint innovation network structure index during 2007–2019 was calculated using Ucinet. Considering the variable calculation of the nodes which entered and exited at any time and combined with the actual situation, the nodes with at least one degree in the network were selected as the empirical research objects, and Stata 12.0 was used for regression analysis to explore how the evolution of innovation network structure impacted crossover innovation performance of emerging technologies. A total of 1,273 sample observed values were finally determined for the regression analysis, including 742 network nodes and 6,731 patents.

#### 4.1.1. Descriptive Statistical Analysis

The analysis results of Table 3 showed that the mean of crossover innovation performance of the dependent variable was 5.288, the standard deviation was 14.091, and the difference between maximum value and minimum value was 195, indicating a large difference in the crossover innovation performance of different emerging technology enterprises. Also, the mean of degree centrality of the independent variables was 0.055, and the standard deviation was 0.196, indicating low differentiation of the network nodes in degree centrality. The mean of the structural hole was 1.397, and the standard deviation was 0.885, indicating a large difference in the structural hole locations occupied by the network nodes. Moreover, the mean and standard deviation of the relationship intensity were 3.623 and 6.73, respectively, which meant that the average amount of cooperation between emerging technology enterprises and innovation partners was about 3.62. The degree of individual differentiation of the enterprises was 6.73, which showed the amount of cooperation between different nodes in the network and innovation partners was greatly different compared with the structural hole. The mean of the network clustering was 2.925, the standard deviation was 17.164, and the extreme deviation was the maximum of all variables, indicating the degree of clustering of the network nodes was greatly different.

#### 4.1.2. Correlation Coefficient Analysis

The analysis results of Table 4 showed there was a significant positive correlation between the structural hole, relationship intensity, and crossover innovation performance, and they passed the test at the 1% significance level. There was a significant positive correlation between the TRT and crossover innovation performance. There was a significant positive linear relationship between the degree centrality of the independent variables, structural hole, and relationship intensity. In addition, there was a correlation between the TRT and independent variables. Considering the influence of multicollinearity among the variables, the Variance Inflation Factor (VIF) of the related variables was analyzed to prevent spurious regression. If the VIF was more than 5, there was collinearity. If the VIF was more than 10, there was serious multicollinearity, which will lead to unstable subsequent model analysis results, and even regression coefficient symbols completely opposite to the actual situation. This indicated that the poorly built model must be handled in time. The analysis results of Table 5 showed that the VIF of all the variables was much less than 5, indicating that there was no multicollinearity among the variables or spurious regression.

### 4.2. Empirical Model Development

According to the analysis results of Table 6, the mean of the crossover innovation performance was 5.29, the variance was 198.556, the skewness was greater than 0, and the kurtosis was much greater than 3, indicating that the sample data for crossover innovation performance clearly did not obey the normal distribution. In addition, the use of OLS estimation will lead to a big error as the patents in the application were not included in the DII, so the linear regression analysis was not applicable to this study. Both the Poisson regression and negative binomial regression are applicable to the case where the dependent variables are discrete nonnegative variables; that is, they describe the probability of a discrete event over a particular time period. However, the Poisson regression requires the data to satisfy the equivalent dispersion; that is, the variance of the dependent variable must be basically equal to the average value. The focused data may cause overdispersion; namely, the mean of the dependent variable is significantly unequal to its variance. Thus, it was more reasonable to use the negative binomial regression because the number of patents belonged to the count type and nonnegative integer. The analysis results of Table 6 showed that the variance was much greater than the mean, indicating highly focused sample data and significant overdispersion. So in this paper, the negative binomial regression was used for empirical analysis.

Based on the above empirical model of the impact of degree centrality (*N*_RD_), structural hole (SH), relationship intensity (NS), and network clustering (NC) on crossover innovation performance (InnoP), the negative binomial regression analysis was conducted. The regression equation was

(4) $$\begin{array}{c} \text{InnoP}=\beta_{0}+\beta_{1}N_{RD}+\beta_{2}SH+\beta_{3}NS+\beta_{4}ND+\beta_{5}\text{TRT}+\beta_{6}N_{Y}+\varepsilon . \end{array}$$

### 4.3. Empirical Analysis Results and Discussion

The regression analysis results are shown in Table 7. The likelihood-ratio test results of M1–M5 and M6 (an overall regression model) were both significant at the 1% significance level, which indicated that they passed the significance test and the dispersion coefficient (*α*) was not 0. And the distribution of the explained variables better satisfied the negative binomial distribution than the Poisson distribution, further indicating that it was appropriate to adopt the negative binomial regression analysis.

The above analysis showed that the network structure had a significant impact on crossover innovation performance, and the correlation between innovation network and crossover innovation performance of emerging technologies was effectively verified. The M6 tested the extent that each network structure variable affected the crossover innovation performance on the whole, suggesting the significant effect of the regression model. The specific analysis results were as follows.

Degree centrality had a positive effect on crossover innovation performance and passed the significance test; hence, the H1 was verified. It means that the enterprises with higher degree centrality will have more nodes for cooperation and communication, along with more opportunities for heterogeneous resources (knowledge, technology, etc.), which enables them to fully utilize the network for crossover technology cooperation for better performance.

Also, the structural hole had a positive effect on crossover innovation performance and passed the significance test; hence, the H2 was verified. That is, the enterprises occupying the structural hole have monopolistic advantages of heterogeneous resources (knowledge, technology, etc.), gaining them easy and full and efficient access and utilization of external resources for crossover technological innovation activities. In this way, the above enterprises can seize the opportunities of technological development and give full play to their technological innovation ability to improve the crossover innovation performance.

Next, relationship intensity had a positive effect on crossover innovation performance at the 1% significance level (*P* < 0.01); hence, the H3 was verified. It indicates that frequent cooperation and communication between enterprises and innovation partners can enhance mutual trust, which facilitates the dissemination of tacit knowledge and the sharing of heterogeneous resources, promoting the crossover innovation and its performance.

However, network clustering had a negative effect on crossover innovation performance and passed the significance test (*β* = −0.005, *P* < 0.05); hence, the H4 was not verified. In other words, under the current trend of emerging technologies, the members of the enterprise with higher network clustering are more likely to work around it to form a small group, then the network structure tends to be a single central one, and the enterprise will carry out technological innovation in several specific technical knowledge fields. Although higher network clustering contributes to deep cooperative innovation, the enterprise with higher network clustering will focus on a more specific field to develop path dependence more easily in partner selection and innovation activities, which limits potential breakthroughs of existing network resources to search for external heterogeneity knowledge and technology crossover cooperation. When a specific field encounters development bottlenecks, further focus on technology R&D and investment will otherwise lead to more decreases in marginal effect of innovation output, thus hindering the improvement of crossover innovation performance.

## 5. Research Conclusion and Implication

This paper analyzed the evolution law of autonomous driving technology innovation network by the SNA with the data on joint applications for relevant invention patents during 2006–2020. The research found as autonomous driving technology developed, and its innovation network showed the evolution characteristics of crossover innovation network of emerging technologies, such as expansion, openness, increasing subjects and enhanced heterogeneity, universal cooperation, diversified knowledge resources, and blurred network boundaries. What is more, the empirical analysis of the relationship between innovation network structure and crossover innovation performance was conducted. The results showed that the innovation network had a significant impact on crossover innovation performance from four aspects, specifically network centrality, structural hole, relationship intensity, and network clustering. Among them, the network centrality, structural hole, and relationship intensity each had a positive effect on crossover innovation performance, while the network clustering had a negative effect. Under the current rapid development of emerging technologies, with greater cooperation and communication ability and potential influence in the network, emerging technology enterprises can upgrade the network centrality, the crossover communication and cooperation, and the existing technological knowledge fields, along with more diversified technological knowledge for better performance. Stronger cooperation and communication among the major innovation-driven entities in the network based on the existing knowledge foundation can also be realized to achieve better performance through in-depth cooperation with other enterprises in relevant fields. More focus on some technological knowledge fields occupying the structural hole for corresponding interactions is to promote the cross-fusion of knowledge in different fields; that is, take advantage of the structural hole in the network to positively promote performance. However, excessively high network clustering on enterprises will have a negative impact. Too centralized network nodes mean too concentrated fields, so enterprises carry out technology R&D and cooperative innovation in some specific knowledge fields, which has a negative impact on the input-output efficiency of innovation, hindering the crossover innovation and its performance.

This paper analyzes the evolutionary pattern of the innovation network of autonomous driving technology and explores the impact of the network structure on crossover innovation performance, but only the cooperative innovation network of patentees was analyzed, only four network structure characteristics variables were studied in relation to the crossover innovation performance of emerging technologies, and there may be other network structure characteristics variables that influence the crossover innovation performance of emerging technologies. Therefore, future studies can expand to other emerging technology fields, and the IPC and patent citation can be included in the study of innovation networks, while the innovation network characteristics variables affecting the crossover innovation performance of emerging technologies can be further explored for more systematic and in-depth analysis.

## Figures and Tables

**Table 1:** Indicators of network topology analysis.

Indicator	Definition
Number of the nodes	Total number of nodes in the network
Number of connecting lines	Total number of links in the network
Network density	Ratio of actual links to all possible links in the network
Average degree	The degree is the sum of the connectivity of a node and the nodes adjacent to it, and the average degree is calculated by dividing the sum of the degrees of all nodes by the total number of nodes in the network
Average path length	Average of the path length between any node pair in the network
Clustering coefficient	The clustering coefficient of a node is the ratio of the actual number of links between neighboring nodes to the maximum possible number of links between them. The clustering coefficient of the network is the average of the clustering coefficients of all nodes

**Table 2:** Indicators of the cooperative innovation network of autonomous driving technology.

Stage	2006–2008	2009–2011	2012–2014	2015–2017	2018–2020
Number of the nodes	39	39	76	202	568
Number of connecting lines	34	33	57	164	495
Average degree	1.744	1.692	1.5	1.624	1.743
Average density	0.046	0.045	0.02	0.008	0.003
Average clustering coefficient	1	0.922	0.925	0.89	0.706
Average path length	1	1.108	1.034	1.155	2.832

**Table 3:** Descriptive statistics of the sample variables.

Variable	Mean	Standard deviation	Minimum value	Maximum value	Observed value
Crossover innovation performance	5.2875	14.0910	1	196	1273
Degree centrality	0.0545	0.1959	0.000	1.873	1273
Structural hole	1.3966	0.8847	1	8.614	1273
Relationship intensity	3.6229	6.7299	1	75.333	1273
Network clustering	2.9265	17.1637	0.000	224	1273
TRT	6.9332	8.3368	2	100	1273

*Note. n* = 742.

**Table 4:** Analysis of correlation coefficients among the sample variables.

	Crossover innovation performance	Degree centrality	Structural hole	Relationship intensity	Network clustering	TRT
Crossover innovation performance	1.000					
Degree centrality	−0.021	1.000				
Structural hole	0.425^*∗∗∗*^	0.017	1.000			
Relationship intensity	0.502^*∗∗∗*^	0.081^*∗∗∗*^	0.310^*∗∗∗*^	1.000		
Network clustering	−0.017	−0.034	0.003	0.015	1.000	
TRT	0.856^*∗∗∗*^	0.013	0.489^*∗∗∗*^	0.501^*∗∗∗*^	−0.016	1.000

*Note. n* = 742, where ^*∗*^*P* < 0.1; ^*∗∗*^*P* < 0.05; ^*∗∗∗*^*P* < 0.01.

**Table 5:** Analysis of VIF of the variables.

	Degree centrality	Structural hole	Relationship intensity	Network clustering	TRT
VIF	1.88	1.34	1.36	1.01	1.61
1/VIF	0.531	0.744	0.738	0.993	0.622

**Table 6:** Descriptive statistics of crossover innovation performance.

Crossover innovation performance	Minimum	Maximum	Mean	Standard deviation	Variance	Skewness	Kurtosis
Effective *N* = 1273	1	196	5.29	14.091	198.556	6.450	54.130

**Table 7:** Results of negative binomial regression analysis for crossover innovation performance.

Variable	Crossover innovation performance (InnoP)					
	M1	M2	M3	M4	M5	M6
*Explanatory variable*						
Degree centrality (*N*_RD_)		0.387^*∗∗*^ (0.178)				0.303^*∗*^ (0.179)
Structural hole (SH)			0.057^*∗∗*^ (0.024)			0.043^*∗*^ (0.024)
Relationship intensity (NS)				0.012^*∗∗∗*^ (0.003)		0.011^*∗∗∗*^ (0.003)
Network clustering (NC)					−0.006^*∗∗*^ (0.002)	−0.005^*∗∗*^ (0.002)

*Control variable*						
Technological resource type (TRT)	0.120^*∗∗∗*^ (0.003)	0.119^*∗∗∗*^ (0.003)	0.117^*∗∗∗*^ (0.003)	0.115^*∗∗∗*^ (0.003)	0.120^*∗∗∗*^ (0.003)	0.112^*∗∗∗*^ (0.003)
Year (*N*_*Y*_)	0.065^*∗∗∗*^ (0.010)	0.084^*∗∗∗*^ (0.013)	0.065^*∗∗∗*^ (0.010)	0.068^*∗∗∗*^ (0.010)	0.066^*∗∗∗*^ (0.010)	0.083^*∗∗∗*^ (0.013)
C	−131.6^*∗∗∗*^ (19.32)	−169.5^*∗∗∗*^ (26.08)	−130.3^*∗∗∗*^ (19.32)	−137.5^*∗∗∗*^ (19.40)	−132.1^*∗∗∗*^ (19.36)	−166.4^*∗∗∗*^ (26.14)
*α*	0.262	0.259	0.260	0.256	0.262	0.253
Log likelihood	−2437.456	−2435.099	−2434.580	−2429.937	−2437.369	−2426.404
LR chi^2	2001.01^*∗∗∗*^	2005.73^*∗∗∗*^	2006.76^*∗∗∗*^	2016.05^*∗∗∗*^	2001.19^*∗∗∗*^	2023.12^*∗∗∗*^
Pseudo *R*^2	0.291	0.2917	0.2919	0.2932	0.2916	0.2942
Likelihood-ratio test of *α* = 0	4427.52^*∗∗∗*^	4320.17^*∗∗∗*^	4419.2^*∗∗∗*^	3955.6^*∗∗∗*^	4420.27^*∗∗∗*^	3864.03^*∗∗∗*^

*Note.* ^*∗*^ *P* < 0.1; ^*∗∗*^*P* < 0.05; ^*∗∗∗*^*P* < 0.01.

## Data Availability

The data used to support the findings of this study are available from the corresponding author upon request.
